# The Interplay Between Stress, Inflammation, and Emotional Attention: Relevance for Depression

**DOI:** 10.3389/fnins.2019.00384

**Published:** 2019-04-24

**Authors:** Viktoriya Maydych

**Affiliations:** Department Psychology and Neurosciences, Leibniz Research Centre for Working Environment and Human Factors, TU Dortmund (IfADo), Dortmund, Germany

**Keywords:** inflammation, cytokines, psychological stress, emotional attention, attentional bias, negative bias, depression, depressive disorders

## Abstract

Depression is among the most significant public mental health issues. A growing body of research implicates inflammation in the etiology and pathophysiology of depression. Yet, the results are somewhat inconsistent, leading to burgeoning attempts to identify associations between components of innate immune system involved in inflammation and specific symptoms of depression, including attention to emotional information. Negative attentional bias, defined as a tendency to direct attention toward negatively valenced information, is one of the core cognitive features of depression and is reliably demonstrated in depressed and vulnerable individuals. Altered attentional processing of emotional information and immunological changes are often precipitated by stressful events. Psychological stress triggers inflammatory activity and affective-cognitive changes that play a critical role in the onset, maintenance, and recurrence of depression. Using various designs, recent studies have reported a positive relationship between markers of inflammation and negative attentional bias on behavioral and neural levels, suggesting that the association between inflammation and emotional attention might represent a neurobiological pathway linking stress and depression. This mini-review summarizes current research on the reciprocal relationships between different types of stressors, emotional attention, inflammation, and depression, and discusses potential neurobiological mechanisms underlying these interactions. The integration provided aims to contribute toward understanding how biological and psychological processes interact to influence depression outcomes.

## Introduction

Depression is a highly prevalent mood disorder in modern society and is associated with significant impairments in the patients’ quality of life. A multitude of basic research and clinical studies have been performed, with the aim of understanding the interaction between biological, psychological, and environmental factors involved in the etiology of depression. There is growing evidence implicating increased levels of markers of inflammation in the pathogenesis of depressive disorders (Raison et al., 2006). Inflammation is a part of the innate immune system’s response to infection or injury. The main mediators of the inflammatory response, proinflammatory cytokines, such as interleukin (IL)-1β, interleukin (IL)-1 receptor antagonist (RA), interleukin (IL)- 6, tumor necrosis factor (TNF)-α, and interferon (IFN)-γ, have been recently shown to communicate with the brain and affect neurotransmission, neuroendocrine activity, and brain structure and functions, thereby inducing emotional, cognitive, and behavioral changes (Haroon et al., 2012). If the inflammatory response remains unresolved, the chronic release of proinflammatory cytokines can promote pathology, including depression. Different studies using a variety of study designs and populations have found positive associations between increased levels of proinflammatory cytokines and symptoms of depression (Dowlati et al., 2010; Valkanova et al., 2013). However, findings have not been entirely consistent for all types of depression (Rothermundt et al., 2001), raising the need for identifying more specific links between inflammation and different somatic and affective-cognitive symptoms, rather than merely testing associations between increased inflammation and categorically defined depression.

According to cognitive models of depression, one of the key features of depressed and vulnerable individuals is biased cognitive processing of emotional and social information. Cognitive biases manifest themselves in a consistent shift toward (self-referential) negative or threatening information in all aspects of cognition, including perception, attention, interpretation, memory, or sensitivity to feedback (Miskowiak and Carvalho, 2014). Negatively biased processing of emotional information is usually regarded both as a neuropsychiatric symptom and as a lingering trait factor that confers cognitive vulnerability to depression and may, when triggered by adverse environmental factors (e.g., stress), initiate the development or reoccurrence of depression (Ingram et al., 1998). The following mini-review focuses primarily on one cognitive domain: attention.

Heightened inflammation and negative attentional bias (AB) are often the results of psychological stress. Acute stressful challenges lead to increases in inflammatory activity and other neurophysiological changes that modulate affective, cognitive, and behavioral processes (Allen et al., 2014; Slavich and Irwin, 2014). Chronic exposure to stressors causes endocrine and immune system dysfunction that contributes to sustained low-grade inflammation, which is involved in the pathogenesis of depressive symptoms (Rohleder, 2014). In parallel, acute stress has been shown to trigger affective and cognitive changes similar to biased information processing characteristic to depression (Gotlib and Joormann, 2010). This evidence has recently led to promising attempts to investigate interactions between emotional attention and inflammation in the context of stress, thereby identifying specific neurocognitive pathways that may be relevant for the etiology of depression and development of novel treatments. The aim of this mini-review is to summarize independent lines of inquiry focusing on the effects of stress (1) on inflammation and (2) emotional attention as well as the potential link between stress-inflammation and stress-cognition pathways (3), and depression.

## Inflammation, Stress, and Depression

A considerable body of evidence suggests that individuals with diagnosed depression exhibit significantly higher levels of IL-1, IL-6, TNF-α, and C-reactive protein (CRP) compared to non-depressed counterparts (Howren et al., 2009; Dowlati et al., 2010). Concurrently, depressive symptoms are more frequent in patients with conditions involving inflammation (e.g., autoimmune diseases) and can be reversed through the use of anti-inflammatory drugs (Kojima et al., 2009; Köhler et al., 2014). Notably, increased inflammatory activity has been documented only in some patients with depression. This indicates that the depression-inflammation link may be modulated by further vulnerability factors, such as genes or cognitive vulnerability. Alternatively, since depression is a heterogeneous disorder, it is also plausible that the association between cytokine-mediated inflammatory processes and depression is more nuanced in terms of the groups of depressive symptoms (somatic vs. affective-cognitive). In support of this notion, a large data set has documented mechanistic links between somatic symptoms of depression and increased inflammation in animals and humans (DellaGioia and Hannestad, 2010); however, studies are lacking in affective-cognitive changes related to inflammatory activity. There is some evidence that inflammatory processes may have differential effects on somatic and affective-cognitive depressive symptoms that are based on distinct neurophysiological mechanisms. For example, studies examining the development of depressive symptoms during the course of IFN-α therapy found that all patients developed somatic symptoms, including fatigue, altered sleep and appetite, motor slowing, during the first weeks of therapy (Capuron and Miller, 2004). In contrast, only 30–50% of patients developed affective-cognitive symptoms such as negative mood, anhedonia, or cognitive impairment during the later stages of therapy. Strikingly, the development of this group of symptoms could be prevented by prophylactic antidepressant administration (Musselman et al., 2001).

There is considerable evidence that psychological stress can activate the inflammatory response. Different types of stressors are capable of eliciting increases in inflammatory activity in a manner that may promote depressive symptoms (Slavich and Irwin, 2014). Moreover, the link between stressor-evoked increases in CRP and proinflammatory cytokines and depression appears to be bidirectional, as chronic stressors and current depressive symptoms, both associated with neurophysiological changes (e.g., glucocorticoid resistance), were found to increase stress reactivity, including cytokine changes in response to stressful challenges.

One of the most robust predictors of increased levels of proinflammatory cytokines is early life adversity (ELA). Usually indicated by parental maltreatment and low socioeconomic status during childhood, ELA is considered as a chronic and severe stressor causing long-lasting psychological and biological abnormalities that considerably increase the risk of depression (Hostinar et al., 2018). Psychological alterations are manifested in exaggerated reactivity to negative information and stress; biological abnormalities include HPA axis activity dysregulation (in most cases hyperactivity leading to glucocorticoid resistance), low parasympathetic activity, and frontolimbic circuit alterations that promote reactivity to threatening stimuli (Callaghan and Tottenham, 2016). For example, individuals exposed to ELA showed stronger increases in proinflammatory cytokines in response to laboratory stress than those who were not (Pace et al., 2006). Moreover, exposure to ELA was prospectively and retrospectively associated with an increased inflammation in later life (Danese et al., 2008; Kiecolt-Glaser et al., 2011; Coelho et al., 2014).

The causal role of stress in inflammatory activity was also examined in laboratory settings that enable the assessment of temporal patterns of cytokine responses and use of standardized stress induction procedures such as the Trier Social Stress Test (TSST) (Kirschbaum et al., 1993; Slavich and Irwin, 2014). Laboratory studies showed that acute stress was associated with significant increases in IL-1β (Yamakawa et al., 2009), IL-1RA and IL-6 (Goebel et al., 2000; O’Donnell et al., 2008; Hackett et al., 2012), and TNF-α (O’Donnell et al., 2008), with IL-β, IL-6, and TNF-α demonstrating the most robust increases (Marsland et al., 2017). At the same time, higher cytokine levels were reported to be associated with increases in negative mood and anxiety in some studies (Yamakawa et al., 2009; Moons et al., 2010; Carroll et al., 2011). The notion that increases in inflammatory activity can lead to negative emotional states was also supported by studies that induced low-grade inflammation through the injection of bacterial endotoxin (i.e., lipopolysaccharide, LPS) or vaccines (i.e., flu, typhoid). Stimulated increases in proinflammatory cytokines were associated with symptoms such as fatigue, negative mood, anhedonia, cognitive impairment, social withdrawal, motor slowing – a variety of symptoms collectively referred to as sickness behavior and resembling those of affective-cognitive and somatic symptoms of depression (Dantzer et al., 2008; Eisenberger et al., 2010). Moreover, the associations between inflammatory activity and sickness behavior were not restricted to the laboratory, but also predicted depressive symptoms and cognitive impairment 1 week later (Kuhlman et al., 2018). Similarly, increases in IL-1β in response to TSST predicted the increase of depressive symptoms 1 year later (Aschbacher et al., 2012). Individuals with diagnosed depression have demonstrated stronger increases in proinflammatory cytokines in response to laboratory stress than non-depressed individuals (Weinstein et al., 2010; Fagundes et al., 2013), indicating an increased inflammatory stress responsiveness in depression. Although the aforementioned studies provide interesting findings, the ecological validity of most results is limited due to laboratory settings and mainly samples of healthy young adults. Future research could seek to examine whether naturalistic stressor-induced increases in proinflammatory cytokines are prospectively associated with depressive symptoms.

## Emotional Attention, Stress, and Depression

Cognitive symptoms of depression include attentional biases (AB) toward negative information (Mathews and MacLeod, 2005). A number of studies using different attention allocation tasks (MacLeod et al., 1986) demonstrated that compared to non-depressed counterparts, depressed individuals exhibit increased difficulty in disengaging their attention from negative stimuli than from positive or neutral stimuli, especially when negative material is related to depression (e.g., feelings of worthlessness, guilt) (Gotlib et al., 2004a,b; Koster et al., 2005; Caseras et al., 2007). Negative AB has been also documented in patients with remitted depression and in individuals exposed to ELA (Luecken and Appelhans, 2005; Joormann and Gotlib, 2007; Raymond et al., 2018).

The common assumption of cognitive stress-diathesis models is that depression is a result of the interaction between cognitive vulnerability and stressful life events (Ingram et al., 1998). Therefore, given cognitive vulnerability, experiencing stressful events can initiate a depressive episode. Although the causal role of stress and cognitive biases that jointly increase the subsequent depression risk is theoretically now well-established, surprisingly few studies have examined this etiological pathway with assessments of stressful events and attention measures that do not rely on the participants’ self-report. A number of laboratory-based studies have examined whether laboratory stress would increase negative AB and whether attention shift would be associated with mood change. Indeed, AB toward negative vs. neutral material has been shown to be increased after a stressful challenge (Ellenbogen et al., 2002; Tsumura and Shimada, 2012). Moreover, attention shift toward negative information was associated with mood lowering (Ellenbogen et al., 2002, 2006) cortisol responses (Ellenbogen et al., 2010; Roelofs et al., 2007) in healthy participants and slower stress recovery in a depressed cohort (Sanchez et al., 2017). Although these results suggest a causal link between stressor-evoked AB and negative mood, the main limitation of this work is that stress effects on AB and mood change reflect short-term prime effects rather than providing ecologically valid evidence of the stress-diathesis hypothesis. To examine the long-term effects, several studies examined whether baseline or stressor-related negative AB shifts would prospectively predict depressive symptoms. These studies reported that AB shift following induction of negative mood interacted with subsequent stressful events in predicting increases in dysphoria 7 weeks later in dysphoric students (Beevers and Carver, 2003). Similarly, negative AB was predictive of the exacerbation of depressive symptoms in adults with subclinical depression after 5 weeks (Disner et al., 2017). Finally, a significant interaction effect of stressful life events and dysfunctional attitudes on clinical depression incidence after 12 months has been reported (Lewinsohn et al., 2001).

## Stress, Inflammation, and Emotional Attention

As outlined in previous sections, distinct lines of research show that different stressors can trigger inflammatory activity and increase the attentional processing of negative information. Both inflammatory processes and cognitive stressor-evoked changes were associated with mood lowering and an increase in anxiety and depressive symptoms. Stressor-evoked elevations in proinflammatory cytokines and attention shift toward negative information can represent stress responses at multiple levels that independently contribute to depressive symptoms. Alternatively, inflammatory and cognitive stress responses may act together, potentiating one another’s impact on promoting depression. The following section provides a summary of studies that examined the relationship between AB and markers of inflammation.

## Behavioral Studies

To analyze an association between negative AB and increased inflammatory activity, several cross-sectional and clinical studies examined performance in attention tasks using emotional material and tested for relationships with inflammatory markers. Levels of CRP were reported to positively correlate with increased AB toward sad vs. happy and angry faces in breast cancer survivors (Boyle et al., 2017). In addition, hepatitis C patients showed an AB away from positive vs. neutral and fearful faces and an increase in symptoms of depression, anxiety, and fatigue 6–7 weeks after commencing IFN-α therapy (Cooper et al., 2018). Concurrently, a greater increase in AB toward self-referential positive vs. negative words and improvement of affective-cognitive and somatic depressive symptoms was observed after completion of anti-TNF-α therapy in patients with inflammatory bowel disease (Gray et al., 2018). This preliminary evidence suggests that affective processing and depressive symptoms may, at least, be partially driven by inflammatory activity. The pattern of AB toward negative and away from positive information is consistent with AB usually observed in depression. However, the influence of disease or environmental confounding factors cannot be ruled out in these studies.

To examine whether a causal relationship exists between stressor-evoked inflammatory response and AB, a number of experimental studies investigated the effects of acute stress or mood induction on cytokine levels and emotional attention in healthy and depressed individuals; however, the findings have been mixed. Significant increases in both pro- and anti-inflammatory cytokines following laboratory stress were reported by some studies (Boyle, 2018; Maydych et al., 2018). Elevations of cytokine levels were, in turn, positively associated with increased AB toward negative and decreased AB toward positive information. While these findings provide support for the notion that stressor-evoked cytokine increases may drive, at least, short-term changes in attention processing with these effects depending on the valence of emotional material, other studies could not confirm this hypothesis (Benson et al., 2017; Niemegeers et al., 2019). The inconsistency of the findings may stem from methodological and design issues, especially from differences in stress/mood induction procedures, the timing of cytokine assessments, and types of attention tasks. It is also plausible that endogenous concentrations of cytokines, in particular in healthy samples, even after a stressful challenge, may be too low to map on behavioral attention measures. Alternatively, attention tasks may not be sensitive enough to the cognitive changes produced by cytokines.

Increased inflammatory activity appears not only to be associated with AB toward negative information but has also been suggested to increase stress reactivity (Dooley et al., 2018). As outlined earlier, depressed individuals and those exposed to ELA exhibit higher increases in proinflammatory cytokines in response to acute stress. Thus, it is possible that exogenously induced inflammation prior to stress manipulation would increase stress reactivity and drive even stronger changes in emotional processing than individual treatments. Increases in IL-6 levels have been demonstrated to be positively associated with negative AB only in response to typhoid vaccine in women with partially remitted depression, but not in response to laboratory stress or both treatments (Niemegeers et al., 2019). In another study, slower processing of negative information was observed in response to LPS treatment and at a trend level in combined LPS and negative mood induction condition in healthy males (Benson et al., 2017).

In summary, the findings from various behavioral studies indicate that stressor-related or endotoxin-induced increases in inflammatory activity may affect emotional attention similar to AB in depression. Yet, there were some inconsistent results and null results, which can reflect methodological differences between studies. In addition, the results obtained in laboratory studies do not allow for conclusions on long-term causal relationships between immune and cognitive processes. Future studies should determine whether increased inflammation can prospectively predict alterations in emotional attention.

## Functional Neuroimaging Studies

Although the literature is rather sparse at present, the effects of inflammation on neural activity and functional connectivity during the processing of emotional stimuli have also been the subject of investigation. The experimental designs of these studies induced increased inflammation through either LPS, vaccines, or laboratory stress and measured neural activity and connectivity during exposure to emotional stimuli or receiving social feedback. LPS-induced inflammation was shown to increase amygdala activity while viewing negative facial expression images (Inagaki et al., 2012). Furthermore, peripheral levels of IL-6 were associated with increased activation of the amygdala and increased functional connectivity between the amygdala and dorsomedial prefrontal cortex (dmPFC) in response to negative social feedback (Muscatell et al., 2015). Laboratory stressor-evoked increases in the soluble TNF-α receptor (sTNFαRII) have been shown to be positively correlated with increased activation in dorsal anterior cingulate cortex (dACC) and anterior insula (AI) in response to social rejection (Slavich et al., 2010; Muscatell et al., 2016). Comparatively, the LPS-stimulated increase in IL-6 was positively associated with increased activation of the bilateral amygdala, dACC, and dorsal prefrontal cortex (dPFC) in response to negative feedback from the confederate based on a 10-min interview previously provided by participants (Muscatell et al., 2016). Other studies reported on the enhanced activity of right inferior orbitofrontal cortex (iOFC) (Kullmann et al., 2013) and subgenual ACC (Harrison et al., 2009) in response to viewing emotional pictures and faces, respectively.

Although the data is not yet sufficient to draw generalized conclusions, the majority of studies have documented the increased activity of amygdala and dACC in response to increases in inflammatory activity and negative social stimuli/feedback. This is consistent with the literature on AB in depressive or at-risk individuals (e.g., those exposed to ELA) that found enhanced and long-lasting activity of amygdala in response to negative material (Disner et al., 2011). A simultaneous activation increase in of PFC and ACC was attributed to cortical insufficiency and abnormal frontolimbic circuit function (Wagner et al., 2006; Matsuo et al., 2007). Along with amygdala and AI, dACC was suggested to constitute a so-called “neural alarm system,” which is responsible for the detection of environmental threats and the regulation of responses to danger including SNS system and HPA axis response (Muscatell and Eisenberger, 2012).

## Mechanisms Linking Inflammation to Emotional Attention

Peripherally released cytokines communicate with the brain and are capable of eliciting changes in emotional processing that mimic affective-cognitive symptoms of depression. Research has identified several pathways by which cytokine signals can access the brain (Haroon et al., 2012). Briefly, cytokines can enter the brain through leaky regions in the blood-brain barrier (e.g., circumventricular organs) or activated monocytes/macrophages recruited to the brain. In addition, cytokine release can be stimulated through brain blood vessel cells (e.g., endothelial cells). Furthermore, afferent vagus nerve fibers can be stimulated to transduce cytokine signals from the periphery into the brain, where the cytokine signals activate cytokine-producing glia cells.

One of the most important molecular mechanisms linking inflammation to emotional cognition is the cytokine effect on the serotonergic system (Capuron and Castanon, 2016). Proinflammatory cytokines activate indoleamine-2,3-dioxygenase (IDO), an enzyme involved in the synthesis of kynurenine from dietary tryptophan. Central and peripheral activation of IDO causes increased catabolism of tryptophan, an important precursor of serotonin, leading to serotonin deficiency (O’connor J. et al., 2009; O’Connor J.C. et al., 2009). Furthermore, the products of kynurenine metabolism, such as quinolinic acid, stimulate the *N*-methyl-D-aspartate (NMDA) receptor, thereby unfolding neurotoxic effects leading to neuronal damage (Campbell et al., 2014).

Another mechanism linking inflammation with cognition is the effect of cytokines on the HPA axis. Cytokines can act on glucocorticoid receptors and indirectly upregulate the synthesis of corticotrophin-releasing hormone (CRH), adrenocorticotropic hormone (ACTH), and cortisol (Raison and Miller, 2003). The extent to which cytokines induce the release of ACTH and cortisol is predictive of the development of affective-cognitive but not of somatic symptoms of depression (Capuron et al., 2003). This implies that HPA axis sensitivity to inflammatory stimulation is particularly relevant for the development of affective-cognitive symptoms of depression.

Finally, the parasympathetic nervous system has been suggested to modulate affective-cognitive and immune processes involved in stress cascade and depression (Thayer and Sternberg, 2006; Ondicova et al., 2010). Lower activity of the vagus nerve is predictive of higher levels of cortisol and cytokine acute stress response (Hamer and Steptoe, 2007; Smeets, 2010; Woody et al., 2017) as well as slower stress recovery (Weber et al., 2010). In addition, the vagal tone has been implicated in the detection and (down-) regulation of inflammatory processes. The anti-inflammatory effects are mediated by the vagal release of acetylcholine, which activates a7 nicotinic Ach receptors in macrophages, thereby inhibiting the release of proinflammatory cytokines from these lymphocytes (the mechanism referred to as “cholinergic anti-inflammatory reflex”) (Rosas-Ballina and Tracey, 2009). It has been also suggested that reduced activity of the vagus nerve is associated with disturbed emotion regulation and further affective-cognitive symptoms that stem from the impaired inhibitory control of the prefrontal cortex over the limbic system (Thayer and Sternberg, 2006; Thayer et al., 2012) as well as deficiency in monoamines (Dorr and Debonnel, 2006).

## Conclusion

In summary, preliminary evidence suggests that acute and chronic stress is associated with increased inflammatory activity and enhanced attentional processing of negative information. Both are predictive of negative mood and depression symptoms that, in turn, increase inflammatory and cognitive stress reactivity. Increased inflammation was associated with a pattern of attentional changes characteristic to depression, whereas affective-cognitive states were predictive of inflammatory stress responses. These findings indicate that immune and affective-cognitive processes are interconnected and may potentiate one another’s impact on depression onset, maintenance or recurrence. An improved understanding of the interplay between inflammatory activity and emotional cognition in the context of stress may help to optimize treatment strategies for depression.

## Author Contributions

VM has conceptualized and written the manuscript.

## Conflict of Interest Statement

The author declares that the research was conducted in the absence of any commercial or financial relationships that could be construed as a potential conflict of interest.
